# 血液病患者第二次异基因造血干细胞移植并发口腔黏膜炎的临床分析

**DOI:** 10.3760/cma.j.cn121090-20240701-00240

**Published:** 2024-12

**Authors:** 晓璐 朱, 景枝 王, 海霞 付, 婷婷 韩, 郑丽 徐, 晓辉 张, 兰平 许, 晓军 黄, 昱 王

**Affiliations:** 北京大学人民医院 北京大学血液病研究所 国家血液系统疾病临床医学研究中心、造血干细胞移植治疗血液病北京市重点实验室，北京 100044 Peking University People's Hospital, Peking University Institute of Hematology, National Clinical Research Center for Hematologic Disease, Beijing Key Laboratory of Hematopoietic Stem Cell Transplantation, Beijing 100044, China

**Keywords:** 造血干细胞移植, 危险因素, 口腔黏膜炎, Hematopoietic stem cell transplantation, Risk factors, Oral mucositis

## Abstract

**目的:**

初步探索接受第二次异基因造血干细胞移植（allo-HSCT）并发口腔黏膜炎的血液病患者的临床特征。

**方法:**

回顾性分析2018年1月至2023年12月于北京大学血液病研究所接受第二次allo-HSCT的58例血液病患者，对照组为1∶2配对的接受第一次allo-HSCT的116例血液病患者。配对因素包括性别、年龄及诊断。比较两组患者口腔黏膜炎发生率及总生存（OS）率。

**结果:**

第二次allo-HSCT组和对照组分别有17例（29.31％）和16例（13.79％）发生口腔黏膜炎（*P*＝0.014），分别有10例（17.24％）和7例（6.03％）发生3级及以上口腔黏膜炎（*P*＝0.019），发生口腔黏膜炎的中位时间分别为移植后第4天（移植后第1～9天）和移植后第5天（移植后第1～10天）。多因素分析结果显示，预处理采用以全身放射治疗为主的方案是发生口腔黏膜炎的独立危险因素（*P*＝0.019）。在发生口腔黏膜炎的患者中，年龄<55岁是发生3～4级口腔黏膜炎的危险因素（*P*＝0.028）。所有接受第二次allo-HSCT的患者均获得粒细胞植入，合并口腔黏膜炎的17例患者粒细胞植活中位时间为移植后14 d，未合并口腔黏膜炎患者粒细胞植活中位时间为移植后12 d，差异无统计学意义（*P*＝0.721）。是否合并口腔黏膜炎不影响急性移植物抗宿主病发生（*P*＝0.938）。第二次allo-HSCT合并与未合并口腔黏膜炎患者的2年OS率差异无统计学意义（51.9％对50.4％，*P*＝0.943）。第二次allo-HSCT合并口腔黏膜炎与第一次allo-HSCT合并口腔黏膜炎患者的2年OS率差异无统计学意义（51.9％对81.3％，*P*＝0.185）。

**结论:**

第二次allo-HSCT患者并发口腔黏膜炎比例显著升高，且程度更为严重。是否合并口腔黏膜炎不影响粒细胞植入、急性移植物抗宿主病发生率和2年OS率。

## Abstract (en)

**Objective:**

This study aimed to investigate the clinical characteristics of oral mucositis（OM）in patients with hematological diseases who received secondary allogeneic hematopoietic stem cell transplantation（allo-HSCT）.

**Methods:**

This study retrospectively analyzed data on 58 patients with hematological diseases who underwent secondary allo-HSCT at the Peking University People's Hospital from January 2018 to December 2023. The control group included 116 randomized patients after primary allo-HSCT during this period（1:2 ratio）with matched gender, age, and diagnosis. The incidence of OM and overall survival（OS）were compared between the two groups.

**Results:**

The secondary allo-HSCT and control groups reported 17（29.31％）and 16（13.79％）cases that developed OM（*P*＝0.014）, whereas 10（17.24％）and 7（6.03％）developed grade ≥3 OM（*P*＝0.019）. The median time for OM to occur was 4 days（1–9 days）and 5 days（1–10 days）posttransplantation in the secondary allo-HSCT and control groups, respectively. The multivariate analysis revealed that the use of whole-body radiation therapy as the main pretreatment regimen was an independent risk factor for OM occurrence（*P*＝0.019）. Among patients with OM, an age of <55 years is a risk factor for developing grade 3–4 OM（*P*＝0.028）. All patients who underwent the secondary allo-HSCT received granulocyte implantation. The median time of granulocyte implantation in 17 patients with OM was 14 days posttransplantation, whereas the median time of granulocyte implantation in patients without OM was 12 days posttransplantation. The difference was not statistically significant（*P*＝0.721）. The presence of OM did not affect the occurrence of acute graft-versus-host disease（*P*＝0.938）. No statistically significant difference was observed in the 2-year OS rate between patients with and without OM during the secondary allo-HSCT（51.9％ *vs* 50.4％, *P*＝0.943）. No statistically significant difference was observed in the 2-year OS rate between patients with OM undergoing the secondary allo-HSCT and those undergoing the primary allo-HSCT（51.9％ *vs* 81.3％, *P*＝0.185）.

**Conclusion:**

The proportion of patients with concurrent OM was significantly increased in the secondary allo-HSCT, and the severity was more severe. Whether or not to merge OM does not affect granulocyte implantation, acute graft-versus-host disease incidence, and 2-year OS rate.

口腔黏膜炎指口腔黏膜上皮炎症性和（或）溃疡性病变，是造血干细胞移植（HSCT）预处理化疗中最常见且最具挑战性的不良反应。口腔黏膜炎主要表现为口腔黏膜充血、红斑、水肿、糜烂及不同程度的溃疡等，覆盖由纤维蛋白、白细胞和上皮碎片组成的假膜，患者常表现为局部疼痛、进食困难、口干及味觉障碍等，且合并真菌、细菌感染风险显著增加，严重影响患者的生活质量，可能导致药物剂量调整或者治疗中止，并增加治疗费用[1]–[2]。

第二次异基因造血干细胞移植（allo-HSCT）是恶性血液病患者第一次HSCT后复发的有效治疗手段。然而，包括口腔黏膜炎在内的多种移植相关并发症仍然是亟待解决的问题。既往文献提示口腔黏膜炎的发生可能与患者的疾病状态、体能状态、预处理方案等因素有关[3]。鲜有文献聚焦第二次移植后口腔黏膜炎发生率及对预后的影响。因此，本研究回顾性比较第二次allo-HSCT与第一次allo-HSCT合并口腔黏膜炎的发生率，并分析发生口腔黏膜炎及其严重程度的危险因素。

## 病例与方法

1. 病例：纳入2018年1月至2023年12月于北京大学血液病研究所接受第二次allo-HSCT的58例恶性血液病患者，对其临床资料进行回顾性分析。对照组为按照1∶2配对的接受第一次allo-HSCT的116例血液病患者，配对因素包括性别、年龄及诊断。本研究经北京大学人民医院伦理委员会批准（伦理批号：2020PHB376-01），所有患者均签署知情同意书。

2. 预处理方案及移植物抗宿主病（GVHD）预防方案：首次移植采用北京大学血液病研究所的常规预处理方案。第二次移植预处理方案采用以全身放射治疗（TBI）为主的方案及以白消安（Bu）为主的方案。首次移植、第二次移植的GVHD预防均采用MMF方案［环孢素A（CsA）+短疗程甲氨蝶呤（MTX）+霉酚酸酯］[4]–[6]，母系或旁系单倍体供者加用低剂量移植后环磷酰胺（PTCy）14.5 mg·kg^−1^·d^−1^，+3 d、+5 d。高龄或造血干细胞移植合并症指数（HCT-CI）≥3的患者应用减低毒性预处理（RTC）方案[7]–[9]。急性GVHD（aGVHD）的诊断和分级见文献[10]–[11]。

3. 植入标准：粒细胞植入指ANC>0.5×10^9^/L，连续3 d。血小板植入指PLT>20×10^9^/L连续7 d且脱离血小板输注。

4. 口腔黏膜炎分级：口腔黏膜炎的分级标准采用WHO口腔毒性量表[12]。0级：口腔黏膜无异常。1级：口腔黏膜有1～2个直径<1.0 cm的溃疡；口腔黏膜出现红斑伴疼痛，但不影响进食。2级：口腔黏膜有1个直径>1.0 cm的溃疡和（或）数个小溃疡；口腔黏膜出现红斑、溃疡，但能进食固体食物。3级：口腔黏膜有2个直径>1.0 cm的溃疡；口腔黏膜出现严重红斑和溃疡，不能进食固体食物。4级：口腔黏膜有2个以上直径>1.0 cm的溃疡和（或）融合溃疡；溃疡融合成片，不能进食固体食物。

5. 随访：采用查阅门诊/住院病历及电话联系等方式进行随访。随访截止时间为2024年5月15日。

6. 统计学处理：数据分析采用SPSS 25.0软件进行。分类变量的组间比较采用*χ*^2^检验或Fisher精确检验，连续变量的组间比较采用*t*检验，连续变量用*M*（范围）表示，分类变量用例数（百分比）表示。口腔黏膜炎的危险因素分析采用单因素和多因素方差分析。总生存（OS）时间定义为供者造血干细胞回输后第1天至随访结束或因任何原因死亡的时间。采用Kaplan-Meier曲线进行生存分析，采用Log-rank检验进行组间比较。双侧*P*<0.05为差异有统计学意义。

## 结果

1. 临床特征：本研究纳入58例接受第二次allo-HSCT的血液病患者，男34例，女24例，中位年龄33（11～59）岁。患者的疾病类型包括：急性髓系白血病（AML）29例，急性淋巴细胞白血病（ALL）19例，骨髓增生异常综合征（MDS）及其他10例。第二次allo-HSCT的预处理方案包括：以TBI为主方案39例（67.2％），以Bu为主方案19例（32.8％）。对照组116例血液病患者的移植预处理方案包括：以Bu为主方案112例（96.5％），以TBI为主方案4例（3.5％），两组患者的临床特征比较见表1。

**表1:** 接受第二次allo-HSCT和第一次allo-HSCT患者的基线临床特征比较

临床特征	第二次allo-HSCT（58例）	第一次allo-HSCT（116例）	*χ*^2^值/*t*值	*P*值
年龄［岁，*M*（范围）］	33（11～59）	34（11～61）	0.363	0.717
性别［例（％）］				1.000
男	34（58.6）	68（58.6）		
女	24（41.4）	48（41.4）		
原发病［例（％）］				1.000
AML	29（50.0）	58（50.0）		
ALL	19（32.8）	38（32.8）		
MDS及其他	10（17.2）	20（17.2）		
移植预处理方案［例（％）］			84.575	<0.001
TBI为主	39（67.2）	4（3.5）		
Bu为主	19（32.8）	112（96.5）		
移植前疾病状态［例（％）］			17.618	<0.001
NR	17（29.3）	7（6.0）		
CR或CRi	41（70.7）	109（94.0）		
MNC输注量［×10^8^/kg，*M*（范围）］	8.5（6.1～15.9）	9.1（7.6～13.5）	0.913	0.343
CD34^+^细胞输注量［×10^6^/kg，*M*（范围））	2.9（1.3～6.6）	2.2（1.1～8.3）	0.686	0.498
造血干细胞来源［例（％）］			0.053	0.818
外周血	39（67.2）	80（69.0）		
外周血+骨髓	19（32.8）	36（31.0）		
单倍体供者［例（％）］			1.275	0.259
是	53（91.4）	99（85.3）		
否	5（8.6）	17（14.7）		
加用低剂量PTCy［例（％）］			13.663	<0.001
是	13（22.4）	5（4.3）		
否	45（77.6）	111（95.7）		
应用RTC方案［例（％）］			3.156	0.076
是	13（22.4）	14（12.1）		
否	45（77.6）	102（87.9）		

**注** allo-HSCT：异基因造血干细胞移植；AML：急性髓系白血病；ALL：急性淋巴细胞白血病；MDS：骨髓增生异常综合征；TBI：全身放射治疗；Bu：白消安；NR：未缓解；CR：完全缓解；CRi：形态学完全缓解而血细胞计数未完全恢复；MNC：单个核细胞；PTCy：移植后环磷酰胺；RTC：减低毒性预处理

2. 口腔黏膜炎发生情况及严重程度分析：58例接受第二次allo-HSCT的血液病患者中17例（29.31％）发生口腔黏膜炎，3级及以上口腔黏膜炎10例（17.2％）。17例口腔黏膜炎患者中男8例，女9例，中位年龄34（16～59）岁，血液原发病包括：AML 9例，ALL 6例，MDS及其他2例。12例采用以TBI为主的第二次移植预处理方案，5例采用以Bu为主的方案。口腔黏膜炎发生的中位时间为移植后第4天（移植后第1～9天）。对照组16例（13.8％）发生口腔黏膜炎，3级及以上口腔黏膜炎7例，与第二次allo-HSCT组相比，差异均有统计学意义（*P*值分别为0.014和0.019）。对照组发生口腔黏膜炎的中位时间为移植后第5天（移植后第1～10天）。

分析口腔黏膜炎发生的危险因素，本研究纳入年龄、性别、基础疾病、移植次数、移植预处理方案、是否应用低剂量PTCy、是否应用RTC方案、是否为单倍体供者，单因素分析显示，移植次数、移植预处理方案的差异有统计学意义（表2）。将单因素分析中*P*<0.2的因素纳入多因素分析，结果显示，采用以TBI为主的预处理方案是发生口腔黏膜炎的危险因素（*P*＝0.019）（表2）。

**表2:** 接受allo-HSCT的血液病患者发生口腔黏膜炎的危险因素分析［例（％）］

因素	口腔黏膜炎		单因素分析		多因素分析	
	有（33例）	无（141例）	*HR*（95％*CI*）	*P*值	*HR*（95％*CI*）	*P*值
年龄			1.630（0.485～5.483）	0.426		
≥55岁	4（12.1）	11（7.8）				
<55岁	29（87.9）	130（92.2）				
性别			1.429（0.667～3.059）	0.357		
男	17（51.5）	85（60.3）				
女	16（48.5）	56（39.7）				
原发病			0.572（0.360～2.259）	0.260		
AML	18（54.5）	69（48.9）				
ALL	13（39.4）	44（31.2）				
MDS及其他	2（6.1）	28（19.9）				
移植次数			2.591（1.196～5.616）	0.014	0.749（0.249～2.128）	0.558
1次	16（48.5）	100（70.9）				
2次	17（51.5）	41（29.1）				
移植预处理方案			3.363（1.510～7.488）	0.002	3.056（1.232～7.576）	0.019
以TBI为主	15（45.5）	28（19.9）				
以Bu为主	18（54.5）	113（80.1）				
移植前疾病状态			2.500（0.966～6.472）	0.053	0.789（0.249～2.493）	0.693
NR	8（24.2）	16（11.3）				
CR或CRi	25（75.8）	125（88.7）				
单倍体供者			0.576（0.206～1.607）	0.288		
是	27（81.8）	125（88.7）				
否	6（18.2）	16（11.3）				
加用低剂量PTCy			1.251（0.384～4.081）	0.710		
是	4（12.1）	14（9.9）				
否	29（87.9）	127（90.1）				
应用RTC方案			1.270（0.468～3.447）	0.639		
是	6（18.2）	21（14.9）				
否	27（81.8）	120（85.1）				

**注** allo-HSCT：异基因造血干细胞移植；AML：急性髓系白血病；ALL：急性淋巴细胞白血病；MDS：骨髓增生异常综合征；TBI：全身放射治疗；Bu：白消安；NR：未缓解；CR：完全缓解；CRi：形态学完全缓解而血细胞计数未完全恢复；PTCy：移植后环磷酰胺；RTC：减低毒性预处理

为进一步分析口腔黏膜炎严重程度的影响因素，比较17例3～4级口腔黏膜炎与16例1～2级口腔黏膜炎患者上述临床特征的差异有无统计学意义。结果显示，年龄<55岁是发生3～4级口腔黏膜炎的危险因素（*P*＝0.028）（表3）。

**表3:** 发生3～4级和1～2级口腔黏膜炎患者临床特征比较［例（％）］

临床特征	3～4级口腔黏膜炎（17例）	1～2级口腔黏膜炎（16例）	*χ*^2^值	*P*值
年龄			4.836	0.028
≥55岁	0（0）	4（25.0）		
<55岁	17（100.0）	12（75.0）		
性别			0.750	0.387
男	10（58.8）	7（43.8）		
女	7（41.2）	9（56.2）		
基础疾病			0.047	0.977
AML	9（52.9）	9（56.3）		
ALL	7（41.2）	6（37.5）		
MDS及其他	1（5.9）	1（6.2）		
移植次数			0.750	0.387
1次	7（41.2）	9（56.2）		
2次	10（58.8）	7（43.8）		
移植预处理方案			0.036	0.849
以TBI为主	8（47.1）	7（43.8）		
以Bu为主	9（52.9）	9（56.2）		
移植前疾病状态			0.010	0.922
NR	4（23.5）	4（25.0）		
CR或CRi	13（76.5）	12（75.0）		
加用低剂量PTCy			0.004	0.948
是	2（11.8）	2（12.5）		
否	15（88.2）	14（87.5）		
应用RTC方案			0.971	0.325
是	2（11.8）	4（25.0）		
否	15（88.2）	12（75.0）		
单倍体供者			0.007	0.935
是	14（82.4）	13（81.2）		
否	3（17.6）	3（18.8）		

**注** AML：急性髓系白血病；ALL：急性淋巴细胞白血病；MDS：骨髓增生异常综合征；TBI：全身放射治疗；Bu：白消安；NR：未缓解；CR：完全缓解；CRi：形态学完全缓解而血细胞计数未完全恢复；PTCy：移植后环磷酰胺；RTC：减低毒性预处理

为进一步分析第二次移植患者口腔黏膜炎发生的危险因素，分析比较58例第二次移植患者中17例发生口腔黏膜炎与41例未发生口腔黏膜炎患者上述临床特征，差异均无统计学意义（*P*值均>0.05）。

3. 口腔黏膜炎与粒细胞植入的相关性：所有接受第二次移植的患者均获得粒细胞植入，合并口腔黏膜炎的17例患者粒细胞植活的中位时间为移植后14 d，未合并口腔黏膜炎患者粒细胞植活的中位时间为移植后12 d，差异无统计学意义（*P*＝0.721）。配对的第一次移植患者均获得粒细胞植入，16例合并口腔黏膜炎患者的粒细胞植活中位时间为移植后15 d，未合并口腔黏膜炎患者的粒细胞植活中位时间为移植后16 d，两组的差异无统计学意义（*P*＝0.863）。

4. 口腔黏膜炎与aGVHD的相关性：本研究纳入的174例患者中59例发生aGVHD，其中11例（18.6％）合并口腔黏膜炎，未发生aGVHD的115例患者中22例（19.1％）合并口腔黏膜炎，两组发生率差异无统计学意义（*P*＝0.938）。58例接受第二次移植的患者中25例（43.1％）发生移植后aGVHD，其中19例（32.8％）发生Ⅱ～Ⅳ度aGVHD。合并口腔黏膜炎的17例患者中6例（35.3％）发生aGVHD，未合并口腔黏膜炎的41例患者中19例（46.3％）发生aGVHD，两组发生率差异无统计学意义（*P*＝0.439）。配对的116例第一次移植患者中34例（29.3％）发生aGVHD，合并口腔黏膜炎的16例患者中5例（31.2％）发生aGVHD，未合并口腔黏膜炎的100例患者中29例（29.0％）发生移植后aGVHD，两组发生率差异无统计学意义（*P*＝0.854）。

5. 口腔黏膜炎对预后的影响：第二次移植后患者的2年OS率为50.8％，合并、未合并口腔黏膜炎患者的2年OS率分别为51.9％和50.4％（*P*＝0.943）。配对的第一次移植患者的2年OS率为77.8％，合并、未合并口腔黏膜炎患者的2年OS率分别为81.3％和77.2％（*P*＝0.738）。第二次移植合并口腔黏膜炎与第一次移植合并口腔黏膜炎患者OS率差异无统计学意义（51.9％对81.3％，*P*＝0.076）（图1）。

**图1:**
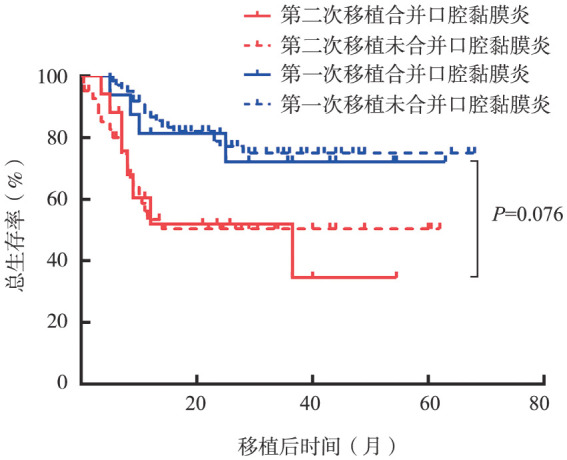
第一次、第二次移植是否合并口腔黏膜炎患者的总生存曲线

## 讨论

本研究显示，接受第二次allo-HSCT的患者并发口腔黏膜炎比例及严重程度显著升高。口腔黏膜炎发生的主要危险因素为第二次移植预处理采用以TBI为主的方案。是否发生口腔黏膜炎不影响粒细胞植入、aGVHD的发生率及患者的OS。

口腔黏膜炎常见的病因或诱因包括化疗、分子靶向治疗、放疗（照射野、剂量和技术）及患者自身的危险因素[3]。既往文献报道，采用以大剂量化疗为主的清髓HSCT预处理方案发生口腔黏膜炎的比例高达70％以上[13]，其中3级以上发生率为67.4％[14]。采用以放射治疗为主的HSCT预处理方案发生口腔黏膜炎的比例高达90％以上[15]。前瞻性研究显示，采用包含大剂量美法仑、Bu和环磷酰胺的预处理方案联合TBI会发生严重的口腔黏膜炎[14],[16]–[17]。

本研究中第一次移植合并口腔黏膜炎的发生率为13.8％，3级及以上口腔黏膜炎的发生率为6.0％。第二次移植患者口腔黏膜炎发生率（29.3％）及3～4级口腔黏膜炎发生率（17.2％）均较第一次移植显著升高。多因素分析结果显示，口腔黏膜炎的发生与以TBI为主的预处理方案有关，而第二次移植中高达67.2％的患者采用了该方案，第一次移植中仅3.5％采用。年龄、性别、基础疾病、加用低剂量PTCy、RTC方案及单倍型供者均未显著影响口腔黏膜炎发生率。由于TBI需在外院进行，且以TBI为主的预处理未能显著降低急性白血病患者第一次完全缓解移植后复发[18]–[19]，故本中心第一次移植较少采用TBI方案。未来可尝试包含靶向药物的增强预处理方案等，减少以TBI为主的预处理方案，降低口腔黏膜炎的发生率。第二次移植患者上述口腔黏膜炎危险因素的差异均无统计学意义，可能与第二次移植前患者体能状态（ECOG评分）、HCT-CI偏高、样本量小等原因相关。口腔黏膜炎严重程度可能与降低强度预处理方案（RIC）、减少剂量（20～35 mg/m^2^）MTX或非MTX的GVHD预防方案有关[20]，但缺乏与RTC相关的研究。本研究对比3～4级口腔黏膜炎与1～2级口腔黏膜炎，结果提示，RTC方案未显著降低3～4级口腔黏膜炎发生率，而年龄<55岁与严重程度相关，与既往文献报道结果相似[21]，推测与年轻患者口腔黏膜上皮细胞分裂速度较快相关，上述未涉及因素仍需进一步研究证实。

预处理方案是口腔黏膜炎发生的重要影响因素，此外，遗传性因素、移植前抗生素的应用也有一定作用。口腔黏膜炎的遗传因素包括调节化疗药物代谢酶活性的基因，如调节叶酸代谢酶活性的基因突变可能导致MTX不良反应增加，患者的黏膜损伤加重[17]。另外，与驱动口腔黏膜炎的生物途径相关的基因表达差异已被证实。与炎症介质（如TNF-α）表达相关的基因多态性与口腔黏膜炎的发生相关[22]。Sonis等[23]发现基于单核苷酸多态性（SNP）的贝叶斯网络，该网络由唾液来源的DNA开发而成，可以预测auto-HSCT后发生口腔黏膜炎的风险。Ye等[24]的研究提示，口腔微生物群与维持口腔稳态和口腔黏膜炎严重程度相关。未来仍需进一步研究口腔黏膜炎的病理生理学，以帮助预测口腔黏膜炎的发生风险[25]。在一篇Cochrane系统评价中，单因素分析结果提示移植前1个月内使用抗生素与严重口腔黏膜炎的发生有关，尽管该因素在多因素分析中差异无统计学意义，但提示微生物菌群在口腔黏膜炎发病中的潜在意义[26]。另有研究发现，接受放疗后并发口腔黏膜炎的头颈部恶性肿瘤患者，其口腔微生物菌群发生改变[27]–[28]，推测其机制可能为共生微生物促进代谢及免疫应答[29]–[30]，但确切机制及其在第二次移植与第一次移植中的差异仍需要进一步探究。

口腔黏膜炎破坏黏膜完整性，可能导致细菌、真菌及病毒等感染增加，推测可能对预后产生潜在影响，尤其是在移植后粒细胞缺乏阶段，但目前尚无定论。Shouval等[21]进行的前瞻性研究纳入115例allo-HSCT患者，结果提示口腔黏膜炎的发生导致粒细胞植入延迟，且消化道感染风险增加。但口腔黏膜炎与GVHD的发生率及OS无关[21]。在本研究中，无论是第一次移植还是第二次移植，口腔黏膜炎不影响粒细胞植入、aGVHD的发生和OS，可能与本中心对口腔黏膜炎进行良好护理、及时的支持治疗、完善的感染预防方案等因素有关。由于本研究为回顾性研究，仍需进一步的前瞻性研究证实口腔黏膜炎与预后的相关性。

本文首次揭示，接受第二次allo-HSCT患者的口腔黏膜炎发生率及严重程度显著增加。临床上应该给予高度重视，控制口腔炎症，缓解疼痛，防治多重感染，促进愈合。在预处理方案中，探索包含靶向药物的增强方案，减少以TBI为主方案，以降低口腔黏膜炎发生率。未来扩大样本的前瞻性临床研究将进一步证实第二次移植的黏膜合并症并探究有效的防治方案。此外，口腔黏膜炎病理生理学机制的深入研究将有助于提出新型有效的预防策略。
